# Differences in the Triglyceride to HDL-Cholesterol Ratio between Palestinian and Israeli Adults

**DOI:** 10.1371/journal.pone.0116617

**Published:** 2015-01-30

**Authors:** Ram Weiss, Hisham Nassar, Ronit Sinnreich, Jeremy D. Kark

**Affiliations:** 1 From the Hebrew University-Hadassah Braun School of Public Health and Community Medicine, Ein Kerem, Jerusalem, Israel; 2 St Joseph Hospital, East Jerusalem and Department of Cardiology, Hadassah University Medical Center, Ein Kerem, Jerusalem; Stellenbosch University, SOUTH AFRICA

## Abstract

**Aims:**

To evaluate differences in the triglyceride to HDL-cholesterol ratio (TG/HDL), thought to be a proxy measure of insulin resistance, between Palestinian and Israeli adults in view of the greater incidence of coronary heart disease and high prevalence of diabetes in Palestinian Arabs.

**Research Methods:**

A population-based observational prevalence study of cardiovascular and diabetes risk factors in Jerusalem. Participants (968 Palestinians, 707 Israelis, sampled at ages 25-74 years) underwent fasting and 2h post-75g oral challenge plasma glucose determinations. Metabolic risk was assessed using the surrogate index TG/HDL. Sex-specific comparisons were stratified by categories of body mass index and sex-specific waist circumference quartiles, adjusted by regression for age, glucose tolerance status and use of statins.

**Results:**

Prevalence of overweight and obesity was substantially larger in Palestinians (p = 0.005). Prevalence of diabetes was 2.4 and 4 fold higher among Palestinian men and women, respectively (p<0.001). Adjusted TG/HDL was higher in Palestinians than Israelis across BMI and waist circumference categories (p<0.001 for both). Higher TG/HDL in Palestinians persisted in analyses restricted to participants with normal glucose tolerance and off statins. Notably, higher TG/HDL among Palestinians prevailed at a young age (25-44 years) and in normal weight individuals of both sexes.

**Conclusions:**

Palestinians have a higher TG/HDL ratio than Israelis. Notably, this is evident also in young, healthy and normal weight participants. These findings indicate the need to study the determinants of this biomarker and other measures of insulin resistance in urban Arab populations and to focus research attention on earlier ages: childhood and prenatal stages of development.

## Introduction

The world is affected by a global rise in the prevalence of obesity. The burden of obesity-driven morbidity is affecting developed as well as developing countries [1]. The mechanism linking obesity with several related co-morbidities such as hypertension, type 2 diabetes and cardiovascular disease is postulated to be obesity-driven peripheral insulin resistance (IR)[2]. The Middle East is also affected by alarming increases in the prevalence of obesity both in children and adults and of diabetes which have been documented in several Arab countries [3, 4]. There is evidence that Israeli Arabs have a higher prevalence of obesity and the metabolic syndrome [5] and diabetes, including an earlier age of onset [6] than Israeli Jews. Urban Palestinian Arabs have been shown to suffer from a greater incidence of coronary heart disease (CHD) [7] and higher CHD mortality than Israelis [8]. The question arises whether there is a specific ethnic predisposition for the development of adverse glucose metabolism and IR in Palestinians that can be demonstrated using a simple biomarker. It has been shown that overweight and obese individuals from specific ethnic backgrounds, such as Hispanics and East Asians, manifest lower insulin sensitivity and greater metabolic risk relative to Whites of similar anthropometric dimensions [9]. Such differences render these individuals prone to develop diabetes and CHD at a lower BMI [10].

The Jerusalem Palestinian-Israeli Risk Factor Study was designed to assess differences in the distribution of cardiovascular risk factors between urban Palestinians and Israelis, following the evidence for substantial disparity in the incidence of myocardial infarction between these two populations [7, 8]. The aim of this report was to compare the triglyceride to HDL-cholesterol ratio (TG/HDL), a recognized biomarker of increased metabolic risk, including of insulin resistance, between Palestinian and Israeli residents of Jerusalem. We postulated that urban Palestinians will exhibit a greater TG/HDL ratio than their Israeli counterparts at comparable BMI and waist circumference levels, and that this will be evident at a young age, consistent with their earlier age of onset of diabetes.

## Methods

The Jerusalem Palestinian-Israeli Risk Factor Study has been previously described [11]. In brief, the study comprises a population-based sample of the city of Jerusalem which since the Six-Day War in 1967 has been unified under Israeli rule. The Palestinians of east Jerusalem have the status of permanent residents of Israel with access to the Israeli job market, national health insurance and social security benefits, and are recorded in the Israel national population registry. Recruitment was performed using an age-sex-stratified random sample of 2000 Palestinian residents from east Jerusalem and 2000 Israeli residents from Jerusalem aged 25–74 drawn from the population registry (200 individuals in each sex-age decile in each population group). Of the Palestinian sample 89.6% were eligible for the study and 29.5% could not be located. The corresponding figures for the Israelis were 88.4% and 25.1%. The response rates for all located eligible residents were 76.7% for Palestinians and 53.7% for Israelis. Participants (970 Palestinians and 712 Israelis) attended two separate examination centers established in east and west Jerusalem for a face-to-face interview and clinical measurements using standardized methods. All participants provided signed informed consent. The study was authorized by the St Joseph Hospital (in east Jerusalem) and Hadassah-Hebrew University Medical Center Ethics (Helsinki) Committees.

Glucose tolerance status was defined as follows: Normal glucose tolerance was defined as a fasting glucose <5.5 mmol/l and a 2-hr glucose <7.7mmol/l, and not on anti-hyperglycemic treatment; pre-diabetes was defined as a fasting glucose *≥*5.5mmol/l and < 6.93 mmol/l or a 2-hr glucose *≥* 7.7mmol/l and below < 11 mmol/l, and not on anti-hyperglycemic treatment; and diabetes was defined as having a documented diagnosis and anti-hyperglycemic treatment or as having a fasting glucose *≥*6.93 mmol/l or a 2-hr glucose *≥*11 mmol/l. The TG/HDL ratio, an established marker of cardiovascular risk [12], was compared between the two ethnic groups.

### Laboratory analyses

All participants had a fasting venous blood sample drawn and those without known diabetes underwent a 2-hour glucose challenge. Blood was immediately separated in a refrigerated centrifuge, aliquoted and stored at-80°C until analysis. Plasma cholesterol, HDL-cholesterol (after precipitation of the apoB-containing lipoproteins with magnesium chloride and phosphotungstic acid) and triglycerides (TG) were measured in the Hadassah University Medical Center lipid research laboratory (that met US-CDC quality control standards) on an auto-analyzer by enzymatic methods using Roche reagents. Plasma glucose was measured using the glucose oxidase method.

### Statistical analysis

Variables are described by means ± standard deviation or as percentages. Variables that were not normally distributed (the TG/HDL ratio and plasma TG) underwent natural logarithmic *(ln)* transformation. Linear regression was performed using the general linear model procedure. The *ln* TG/HDL ratio was introduced as the dependent variable, with ethnic group as an independent variable, adjusting for age, sex, BMI (categorical), waist circumference (continuous), glucose tolerance status (categorical) and use of statins in a series of models. In parallel to the ethnic group comparisons across BMI categories, we also modeled ethnic group differences across sex-specific waist circumference quartiles. Analyses were repeated for subsets of participants with normal glucose tolerance and not on statins and for the younger group aged 25–44. Analyses were performed using SPSS19.0 for Windows.

## Results

### Characteristics of the study participants (Table 1)

Participants with complete data, 968 Palestinians (516m/452f) and 707 Israelis (370m/337f), were included in the analysis. Israeli men and women were taller than their Palestinian counterparts. Palestinian men and particularly women had a greater mean body mass index (BMI) than the Israelis (p = 0.02 and p<0.001, respectively). These differences are reflected in the prevalence of overweight and obesity (p = 0.005 and p<0.001 for men and women, respectively), that was especially large in Palestinian women, with 57% being classified as obese vs. 30% of Israeli women, as compared with 31% of Palestinian men and 22% of Israeli men. Only 13% of Palestinian women were classified as normal weight vs. 35% of Israeli women. Waist circumference was larger in Palestinian compared to Israeli females (p<0.001) and was above the metabolic syndrome definition threshold of 88cm in 74% of Palestinian women and 51% of Israeli women. The excessive obesity of Palestinian women is supported by highly significant interactions of sex and ethnic group with BMI and waist circumference (P<0.001). The prevalence of diabetes was 2.4 and 4-fold higher in Palestinian men and women, respectively, in comparison to their Israeli counterparts (pχ^2^<0.001 for both sexes). Statin use was generally similar between Palestinians and Israelis. Plasma total cholesterol and LDL-cholesterol levels were similar between Palestinians and Israelis, whereas triglyceride levels were higher and HDL-cholesterol levels were overtly lower in Palestinian men and women (p<0.001 for both). Importantly, ln TG/HDL was substantially higher in Palestinian men and women in comparison to their Israeli counterparts (P<0.001 for both), and was higher in men than women in both population groups

**Table 1:** Characteristics of Palestinian and Israeli study participants.

	**Israeli (n = 707)**		**Palestinian (n = 968)**		**Significance**
	**Males** **(n = 370)**	**Females (n = 337)**	**Males** **(n = 516)**	**Females (n = 452)**	
**Age (yrs)**	51.6±13.9	53.3±13.1	51.5±14.1	51.8±13.9	NS
**Height (cm)**	172±7	159±6	171±7	156±6	P = 0.005 for PM vs. IM P<0.001 for PF vs. IF
**Weight (Kg)**	81.6±14.6	70.7±15.3	82.2±15.0	77.1±15.4	P<0.001 for PF vs. IF
**BMI (Kg/m^2^)**	27.3±4.2	27.8±5.8	28.0±4.7	31.7±6.4	P = 0.02 for PM vs. IM P<0.001 for PF vs. IF
**Weight category (%)** **(N/OW/OB)**	29/49/22	35/35/30	26/43/31	13/30/57	pχ^2^ = 0.005 for PM vs. IM pχ^2^<0.001 for PF vs. IF
**Waist circumference (cm)**	98±12	89±14	98±13	97±14	P<0.001 for PF vs. IF
**Glucose tolerance** **(NGT/PRE-D/DM) (%)**	36/54/10	52/42/6	32/44/24	35/40/25	pχ^2^<0.001 for PM vs. IM and for PF vs. IF
**Statin Use (N/Y) (%)**	84/16	76/24	78/22	75/25	pχ^2^ = 0.04 for PM vs. IM
**Plasma Cholesterol (mmol/l)**	4.61±0.82	4.86±0.88	4.58±0.98	4.73±1.01	NS
**Plasma LDL-cholesterol (mmol/l)**	2.90±0.72	2.92±0.75	2.84±0.85	2.82±0.90	NS
**Plasma HDL-cholesterol (mmol/l)**	1.06±0.28	1.34±0.33	0.95±0.23	1.19±0.33	P<0.001 for PM vs. IM P<0.001 for PF vs. IF
*Plasma Triglycerides (mol/l)	1.19[0.85–1.77]	1.09[0.82–1.49]	1.42[0.99–2.07]	1.32 [0.93–1.94]	P<0.001 for PM vs. IM P<0.001 for PF vs. IF
*TG/HDL	2.67 [1.78–4.29]	1.80 [1.27–2.90]	3.38[2.19–5.58]	2.68 [1.62–4.26]	P<0.001 for PM vs. IM P<0.001 for PF vs. IF

The values presented are means and SDs or proportions.

Comparisons by sex between ethnicities were performed using a Student t test or a χ^2^ test where indicated.

*Triglycerides and TG/HDL ratio were not normally distributed and are presented as medians and inter-quartile ranges

Weight categories: N-normal weight (BMI<25kg/m^2^), OW—overweight (25< BMI<30 kg/m^2^); OB—obese—(BMI 30+ kg/m^2^)

Glucose tolerance categories: NGT-normal glucose tolerance (fasting plasma glucose<5.5 mmol/ll and 2-hr plasma glucose<7.7mmol/l); PRE-D- Impaired fasting glucose [5.5> FPG<6.93mmol/l or impaired glucose tolerance (2-hour glucose 7.7–11.0 mmol/l)]; DM- Diabetes (on anti-hyperglycemic medications due to diagnosis of diabetes or fasting glucose >5.5mmol/ll or 2-hour glucose >11.0mg/dl).

### Determinants of TG/HDL in Israeli and Palestinian Adults (Table 2)

We evaluated the determinants of *ln* TG/HDL by a series of regression models. Palestinians showed significantly higher *ln* TG/HDL (B = 0.30, p<0.001) in an unadjusted analysis (model 1). As both sex and age were associated with *ln* TG/HDL (r = 0.062, p = 0.01 for age), they were included in the model (model 2). This adjustment did not affect the association with ethnic background and affirmed that men had substantially higher TG/HDL than women (p<0.001) and that age was positively associated with TG/HDL. Adding the degree of obesity, glucose tolerance (as defined in Table 1) and use of statins (model 3) confirmed that obesity and overweight were strongly associated with greater *ln* TG/HDL (B = 0.46, p<0.001 for obese vs. normal weight, B = 0.28, p<0.001 for overweight vs. normal weight) and reversed the association of age with TG/HDL. As expected, normal glucose tolerance was associated with lower *ln* TG/HDL (B=-0.29, p<0.001 for NGT vs. diabetes and B=-0.12, p = 0.001 for NGT vs. pre-diabetes) and statin use was associated with greater *ln* TG/HDL (B = 0.15, p<0.001). Inclusion of these covariates in the model attenuated the association with population group (B = 0.18 for Palestinians vs. Israelis, p<0.001), but indicated that only part of the difference between the population groups in TG/HDL is attributable to current obesity or overweight. Addition of waist circumference to the model (model 4) did not affect the association of ethnic group with TG/HDL (B = 0.19, p<0.001). Tests for two-way interactions of sex and ethnicity (p = 0.54), BMI and ethnicity (p = 0.17), and age and ethnicity (p = 0.11), and the three-way interaction of sex, BMI, and ethnicity (p = 0.15) were all non-significant. Restriction of the analysis to subjects with normal glucose metabolism and off statins (576 participants, models 5–7) yielded almost identical results for the association of ethnic group with *ln* TG/HDL, whereas the inverse association with age was attenuated.

**Table 2:** Determinants of the ln TG/HDL-cholesterol ratio in Israelis and Palestinians.

	**All Participants (n = 1675)**												**NGT + Off Statins (n = 576)**											
	**Model 1**			**Model 2**			**Model 3**			**Model 4**			**Model 5**			**Model 6**			**Model 7**		
	**B**	**SE**	**P**	**B**	**SE**	**P**	**B**	**SE**	**P**	**B**	**SE**	**P**	**B**	**SE**	**P**	**B**	**SE**	**P**	**B**	**SE**	**P**
**Group** **(Palestinian = 1, Israeli = 0)**	0.30	.03	**<0.001**	0.30	0.03	**<0.001**	0.18	0.03	**<0.001**	0.19	0.03	**<0.001**	0.27	0.06	**<0.001**	0.17	0.05	**0.001**	0.18	0.05	**<0.001**
**Age (yrs)**				0.004	0.001	**0.001**	-0.003	0.001	**0.01**	-0.005	0.001	**<0.001**				-0.002	0.002	**0.41**	-0.004	0.002	0.07
**Sex (M = 1, F = 0)**				0.31	0.03	**<0.001**	0.34	0.03	**<0.001**	0.25	0.03	**<0.001**				0.42	0.05	**<0.001**	0.31	0.06	**<0.001**
**BMI Category** **Obese vs normal weight**, **Overweight vs. normal weight**							0.46 0.28	0.04 0.04	**<0.001** **<0.001**	0.16 0.15	0.07 0.05	**0.012** **0.001**				0.50 0.38	0.07 0.06	**<0.001** **<0.001**	0.18 0.24	0.12 0.07	0.12 **0.001**
**Glucose Tolerance** **DM vs NGT** **IGT vs NGT**							0.29 0.12	0.05 0.04	**<0.001** **0.001**	0.25 0.10	0.05 0.04	**<0.001** **0.007**									
**Statin Use (Y = 1, N = 0)**							0.15	0.04	**<0.001**	0.15	0.04	**<0.001**									
**Waist Circumference (cm)**										0.012	0.002	**<0.001**							0.012	0.003	**0.001**
**Adjusted R^2^**	**0.044**			**0.096**			**0.200**			**0.217**			**0.040**			**0.217**			**0.232**		

B’s are un-standardized regression coefficients.

In order to limit possible effects of aging and comorbidity and to assess whether the ethnic association is already evident in young adults, we restricted the analysis to participants aged 25–44 years (348 Palestinians,185m/163f and 224 Israelis, 134m/90f) (not shown, but parallel to model 4 in table 2). Greater TG/HDL was observed in Palestinians compared to Israelis in both sexes (sex-adjusted B = 0.20, p<0.001). Further restricting the analysis in this age group to those with normal glucose tolerance and off statins (189 Palestinians, 94m/95f and 143 Israelis, 74m/69f) (not shown, but parallel to model 7) yielded consistent results for Palestinians vs. Israelis (B = 0.17, p = 0.001).

### TG/HDL by BMI and waist circumference categories (Figs. 1 and 2)

**Figure 1:**
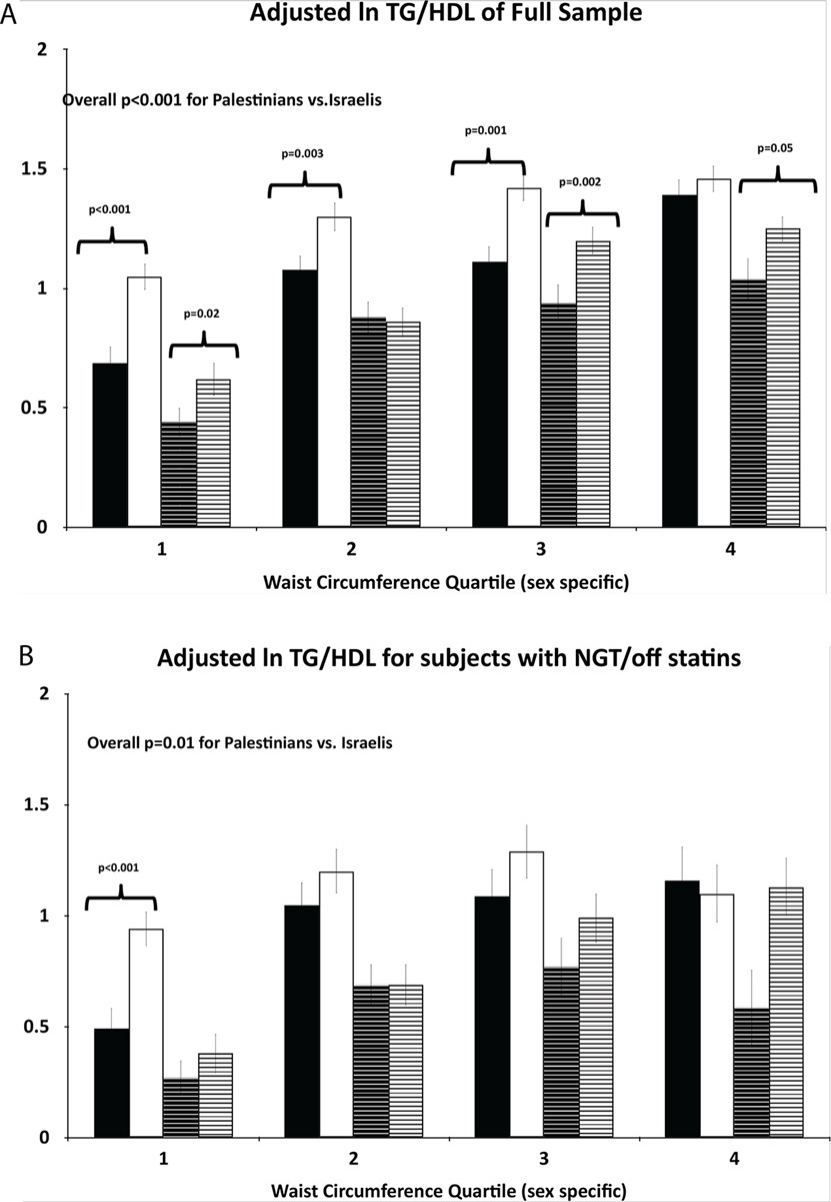
Association of the TG/HDL ratio with population group (Palestinians, Israelis) by sex across BMI categories. Upper panel (A): In the full sample adjusted by linear regression in a model with the following independent variables—ethnic group, BMI category, sex, age, glucose tolerance status, and use of a statin. Lower panel (B): In the sample restricted to those with normal glucose tolerance and off statins, adjusted in a model that included ethnic group, BMI category, sex and age. The presented p value is for the Palestinian-Israeli differences in the TG/HDL ratio. Bars: Black—Israeli males, white—Palestinian males, Black dashed—Israeli females, white dashed—Palestinian females (data are presented as means±SEs).

**Figure 2:**
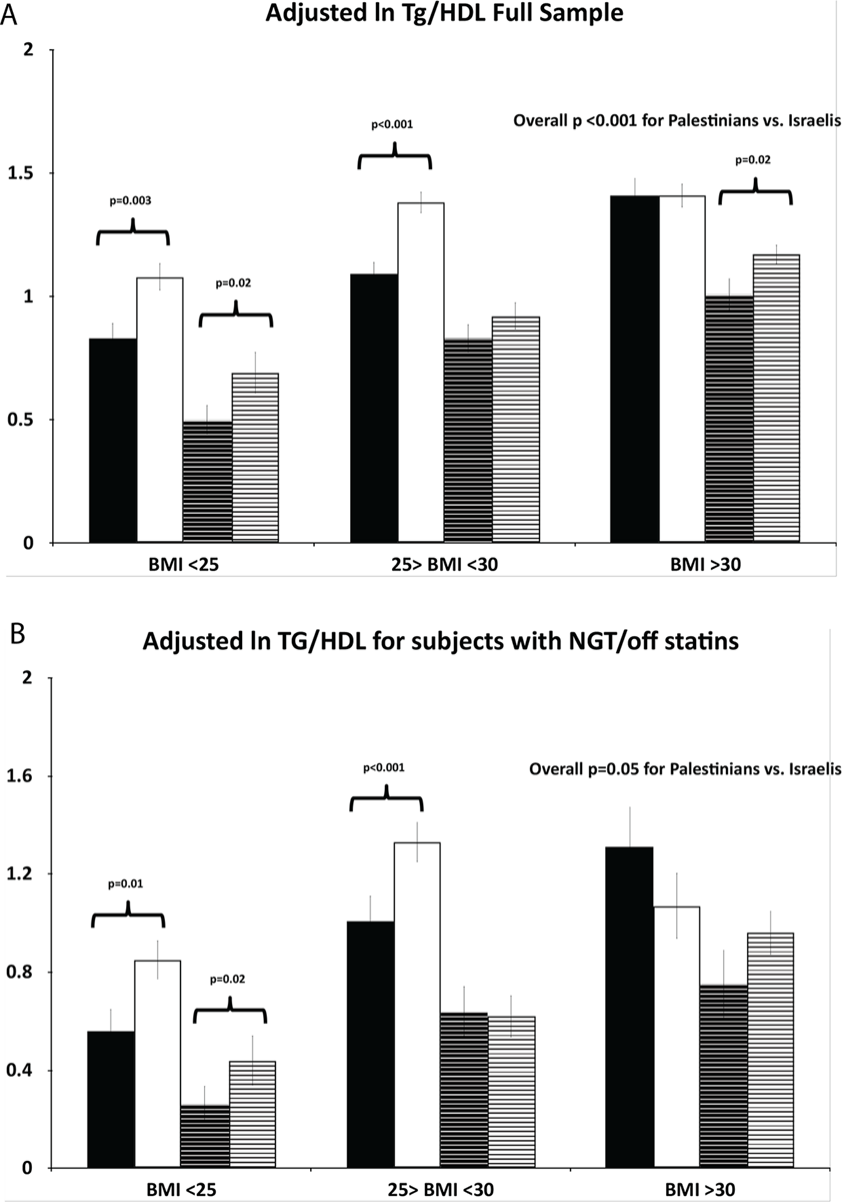
Association of the TG/HDL ratio with population group by sex across waist circumference quartiles. Upper panel (A): In the full sample adjusted by linear regression in a model with the following independent variables—ethnic group, waist circumference (WC) quartiles, sex, age, glucose tolerance status, and use of a statin. Lower panel (B): In the sample restricted to those with normal glucose tolerance and off statins, adjusted in a model that included ethnic group, WC quartiles, sex and age. The presented p value is for the trend of the TG/HDL ratio by WC categories. Bars: Black—Israeli males, white—Palestinian males, Black dashed dashed—Israeli females, white dashed—Palestinian females (data are presented as means±SEs). Male WC quartiles: 1: <90cm; 2: 90 ≤ and <97cm; 3: 97≤and <105cm; 4: ≥105cm. Female WC quartiles: 1: <82cm; 2: 82≤ and < 93; 3: 93≤ and <103; 4: ≥ 103cm.

In order to determine whether TG/HDL ratios are higher in Palestinians than Israelis of normal weight and girth, we assessed sex-specific ethnic differences in *ln* TG/HDL across three BMI categories: normal weight (BMI<24.99 kg/m^2^), overweight (BMI *≥*25.0 and <30.0 kg/m^2^) and obese (BMI*≥*30.0 kg/m^2^) (Fig. 1) as well as across sex-specific quartiles of waist circumference (Fig. 2), each adjusted for age, glucose tolerance status and use of statins. We observed that Palestinians had substantially higher TG/HDL than Israelis in the normal weight group in both sexes in the full sample (p = 0.003 and p = 0.02 for men and women, respectively) (Fig. 1, upper panel), that persisted when the analysis was restricted to the smaller sample of those with normal glucose tolerance and off statins (p = 0.01 and p = 0.02 for men and women, respectively) (Fig. 1, lower panel). Similarly, Palestinians in the lowest waist circumference quartile displayed significantly greater TG/HDL than Israelis (Fig. 2).

Overall, in both sexes, across the BMI categories (with the exception of obese men in the NGT-off statin group, Fig. 1, lower panel) and across the waist circumference categories (with the exception of men in the upper quartile of the NGT-off statin group, Fig. 2, lower panel) the TG/HDL ratio was higher in Palestinians than Israelis. A significant 3-way interaction of sex, ethnicity and BMI (p = 0.012) supporting an opposite ethnic association in obese men was restricted to the NGT-off statin ‘healthy” group (Fig 1, lower panel); none of the other 3-way interactions or any of the 2-way interactions were statistically significant.

Thus, per given degree of obesity and waist circumference, Palestinians of both sexes displayed a generally greater TG/HDL than their Israeli counterparts. These effects were pronounced in the normal weight and lowest waist circumference categories.

## Discussion

Palestinians exhibited a substantially greater prevalence of DM than Israelis in our study, consistent with findings in Israeli Arabs [6]. Furthermore, Palestinians have been shown to have greater CHD incidence [7], CHD mortality [8] and stroke mortality (the latter in Israeli Arab citizens) [13] in comparison to Jewish Israelis. As the burden of non-communicable diseases is on the rise across the Arab world [14], it is crucial to identify the underlying determinants of this trend as well as the more proximal mechanistic pathophysiological explanations that may be relevant for design of preventive interventions for Arabs in general with specific emphasis in this case for Palestinians [15]. In the latter, diabetes and cardiovascular disease are recognized as high priority targets for preventive intervention [16]. We show that per given BMI or waist circumference category, and remarkably among the normal weight subjects, even after adjustment for age, sex, glucose tolerance status and use of statins, Palestinians have higher TG/HDL in comparison to Israelis. These findings generally persisted when the analysis was restricted to individuals with ostensibly normal glucose metabolism and off statins. Importantly, we show that even young adult Palestinians demonstrated increased multivariable-adjusted TG/HDL.

In both the BMI and waist circumference analyses, obese men (but not women) showed either no association or an inverse association of ethnicity with TG/HDL. The 3-way interaction, which captures this situation, was significant in the BMI analysis only in the healthy subgroup, which differs from the full sample in the exclusion of abnormal glucose metabolism and statin-treated dyslipidemia. As we had no prior hypothesis in this regard, we cannot exclude chance as an explanation for this finding which implies that obesity in men overwhelms the impact of ethnicity that is evident in the lower BMI range.

Arabs and Jews in Israel show a persistent gap in life expectancy that has been maintained since the mid-70s. Part of this gap is explained in recent years by a greater mortality due to cardiovascular disease and type 2 diabetes [17]. While specific risk factors such as a greater prevalence of cigarette smoking in men [18], lower physical activity and obesity [19] have been shown in Palestinians, our findings suggest that independent of increased obesity, i.e. across BMI and waist circumference categories, the Palestinians display greater TG/HDL. This biomarker has been shown in several populations to associate with insulin resistance [20]– the major “driving force” of most if not all components of the metabolic syndrome [21]. Moreover, upon examination of sex-specific quartiles of waist circumference, a stronger correlate of abdominal lipid partitioning [22], Palestinians of both sexes demonstrated greater TG/HDL per given waist category. This excess in TG/HDL relative to Israelis was already present in young adulthood, consistent with an earlier age at onset of diabetes [7], and was evident in those of normal weight and in individuals without altered glucose metabolism and off statin therapy. Putative explanations for this phenomenon may include relatively lower amounts of lean body mass, altered number or function of skeletal muscle mitochondria and potentially inherent differences in elements of the insulin signal transduction pathway [23].

When comparing Israelis and Palestinians of similar BMI categories, Palestinians have greater TG/HDL (and thus by inference may be more insulin resistant), even after adjustment for waist circumference. A potential explanation for this observation may be differences of total body fat and patterns of lipid partitioning such as hepatic and intra-myocellular lipid accumulation [24] that were not evaluated in this analysis. While waist circumference serves as a surrogate for visceral fat, differences in intra-myocellular and hepatic lipid deposition may have a major impact on whole body insulin sensitivity and may explain some of our findings [25, 26]. It has been previously shown that intra-myocellular fat deposition is ethnicity dependent; thus Palestinians, similar to Hispanics, may favor lipid deposition in the muscular compartment that can lead to increased insulin resistance and cardiovascular risk. Visceral fat is known to correlate with intra-hepatic fat deposition. We show that per given sex-specific waist circumference, Palestinians exhibit a greater TG/HDL, a correlate of greater insulin resistance and a predictor of CVD risk. As differences in TG/HDL were already evident in young adults, one can hypothesize that differences in the relation of visceral and hepatic lipid deposition and hepatic insulin sensitivity may exist between Israelis and Palestinians. Another option that deserves further investigation is that per given waist circumference, Palestinians show a stronger association with visceral fat content. It has recently been shown that first trimester fetal growth restriction is associated with increased IR at the age of six years [27] suggesting that such differences may begin in-utero, possibly by inducing specific epigenetic profiles. Indeed, epigenetic changes in peripheral blood leukocytes at the FTO locus are associated with increased risk of diabetes in Israelis [28], a finding that has been confirmed in our Palestinian sample (Toperoff G, Kark JD, Nassar H, et al, in preparation).

The advantages of this analysis include the reasonably large population-based sample drawn from urban-dwelling Israelis and Palestinians across five decades of the lifespan and the dearth of such studies in the past.

Study limitations include the cross-sectional design and the possibility of selection bias due to possible differential nonresponse in the sample. If so the study sample might not fully represent the source population in terms for example of the proportions of diabetics. However, on restriction of the analysis to those free of clinically-impaired glucose metabolism and off statins the findings persisted largely unaltered. An additional limitation is that insulin resistance was not directly measured in this study. However, TG/HDL is well correlated with clamp-derived measures of insulin sensitivity in some studies and with HOMA-IR in others and is thus considered to be an informative surrogate of whole body insulin sensitivity [29]. Furthermore, the TG/HDL ratio that has been quite widely used in epidemiological studies has proven to be a good predictor of adverse cardiovascular outcomes in a number of populations [30]. A further limitation is the lack of adjustment for health-related behaviors such as physical activity, cigarette smoking, and dietary practices. These factors affect plasma lipids as well as insulin sensitivity and may play a role in explaining TG/HDL ratio differences between the two groups.

Our findings highlight an important difference between Palestinians and Israelis in the levels of an established cardiovascular risk indicator and a potential surrogate of insulin resistance, the TG/HDL ratio. The source of this difference that was independent of age, degree of obesity, waist circumference, glucose tolerance status and statin use remains unclear as yet. Further studies focusing on lipid deposition patterns and carbohydrate and lipoprotein metabolism in these populations are warranted. Preventive measures against the development of cardiovascular disease and type 2 diabetes in Palestinians, as well as research into the age of onset and determinants of insulin resistance, should thus begin in adolescence, childhood and perhaps in infancy and the prenatal period.
